# Associations Between Segmental Upper-Extremity Isometric Strength and Shuttlecock Velocity in Young Badminton Players

**DOI:** 10.3390/jfmk11030360

**Published:** 2026-09-09

**Authors:** Yalcin Aydin, Varol Tutal, Fatma Beyza Bilgic, Aslı Ceyhan, Eda Bayer, Cíntia França, Fahri Safa Cinarli

**Affiliations:** 1 Faculty of Health Sciences, Malatya Turgut Ozal University, Malatya 44900, Türkiye; yalcin.aydin@ozal.edu.tr (Y.A.); varol.tutal@ozal.edu.tr (V.T.); 2 Faculty of Sport Sciences, Department of Physical Education and Sports Teaching, Batman University, Batman 72100, Türkiye; fatmabeyza.bilgic@batman.edu.tr; 3 Malatya Provincial Directorate of Youth and Sports, Malatya 44100, Türkiye; asli_cceyhan@hotmail.com; 4 Department of Physical Education and Sports, Inonu University, Malatya 44280, Türkiye; edabayer15@gmail.com; 5 Department of Physical Education and Sport, University of Madeira, 9020-105 Funchal, Portugal; cintia.franca@staff.uma.pt; 6 Laboratory for Robotics and Engineering Systems, Interactive Technologies Institute, 9020-105 Funchal, Portugal; 7 Department of Movement and Training Science, Inonu University, Malatya 44280, Türkiye

**Keywords:** badminton, velocity, upper-extremity strength, athletic performance

## Abstract

**Background and Objectives**: Upper-extremity strength is considered an important contributor to badminton stroke performance; however, the independent contributions of specific muscle groups to shuttlecock velocity in young players remain unclear. This study investigated the associations between segmental upper-extremity isometric strength and shuttlecock velocity in young badminton players. **Methods**: Twenty-four competitive U11 badminton players (12 males and 12 females; mean age, 10 years) participated in this cross-sectional study. Handgrip and segmental upper-extremity isometric strength (elbow, shoulder, and wrist muscle groups) were assessed using dynamometry. Shuttlecock velocity during a badminton smash was measured using a radar gun. **Results**: Shuttlecock velocity was positively correlated with all measured upper-extremity strength variables (*r* = 0.408–0.704, all *p* < 0.05). After adjustment for sex and upper-limb length, only shoulder extension (*r* = 0.529, *p* = 0.011), shoulder flexion (*r* = 0.557, *p* = 0.007), shoulder external rotation (*r* = 0.430, *p* = 0.046), and shoulder internal rotation strength (*r* = 0.495, *p* = 0.019) remained significantly associated with shuttlecock velocity. Multiple regression analyses confirmed these shoulder-specific strength measures as independent predictors of shuttlecock velocity (β = 0.343–0.468, all *p* < 0.05), explaining 57.2–63.8% of the adjusted variance. **Conclusions**: Although shuttlecock velocity was initially associated with all upper-extremity strength measures, only shoulder-specific strength remained associated with performance after adjustment for sex and upper-limb length. These exploratory findings suggest that shoulder strength may represent a relevant physical correlate of badminton smash performance in young athletes; however, confirmation in larger, adequately powered studies is required.

## 1. Introduction

Badminton is one of the fastest racket sports in the world, and shuttlecock velocity during a smash represents one of the most distinctive characteristics of high-level performance. Previous studies have shown that smash velocity increases progressively with playing expertise, rising from approximately 108 km·h^−1^ (30 m·s^−1^) in young players and nearly 252 km·h^−1^ (70 m·s^−1^) in elite athletes, with velocity improvements occurring in a largely linear manner across skill levels [1,2]. In elite international players, shuttlecock velocities exceeding 288 km·h^−1^ (80 m·s^−1^) have also been reported during smash actions [3]. Beyond its biomechanical relevance, shuttlecock velocity is strongly associated with competitive performance. Previous research has demonstrated significant relationships among shuttlecock velocity, player ranking, competitive level, tournament point accumulation, and offensive effectiveness during match play [4]. Indeed, shuttlecock velocity has been identified as one of the strongest predictors of badminton skill level and competitive success, highlighting its importance as a key performance indicator [5].

The generation of high shuttlecock velocities is a complex movement task involving the coordinated contribution of multiple body segments through a proximal-to-distal kinetic chain. Biomechanical studies have consistently demonstrated that successful smash performance relies on the sequential transfer of energy from the trunk and shoulder to the more distal segments of the upper extremity in a whip-like motion pattern [6,7]. Within this sequence, trunk rotation contributes to the generation of angular momentum and facilitates efficient energy transfer through the kinetic chain, whereas shoulder internal rotation, elbow extension, and wrist flexion play critical roles in accelerating the racket immediately prior to shuttlecock contact [7,8]. Recent evidence has further shown that greater pelvis–thorax separation, shorter acceleration phase duration, and greater shoulder internal rotation at impact are associated with higher shuttlecock velocities, highlighting the importance of coordinated proximal and distal segment actions during smash execution [9]. Although shuttlecock velocity is influenced by multiple technical and biomechanical factors, muscular strength remains one of the most modifiable physical qualities through training and therefore represents an attractive target for performance enhancement programs.

Upper-extremity strength is believed to play a particularly important role in badminton, given the high demands placed on the shoulder, elbow, wrist, and hand during stroke production. Evidence from overhead racket sports suggests that greater upper-extremity strength is associated with higher stroke velocities. In competitive tennis players, shoulder-specific force production characteristics, particularly shoulder internal rotation and shoulder flexion strength, have been identified as important determinants of serve velocity performance [10,11]. Similarly, isokinetic shoulder strength has demonstrated moderate to very strong associations with serve velocity, with shoulder flexion, extension, abduction, and internal rotation among the strongest predictors of ball speed [12]. In youth tennis players, upper-body strength indicators such as handgrip strength have also been associated with serve and groundstroke velocities [13]. Collectively, these findings suggest that upper-extremity strength contributes meaningfully to overhead stroke performance. Nevertheless, the contribution of specific upper-extremity muscle groups to shuttlecock velocity remains insufficiently understood. Most previous studies have focused on general strength characteristics, overall performance measures, or athletes from other racket sports, providing limited information regarding the relative importance of individual upper-extremity segments in badminton.

This limitation may be particularly relevant in youth badminton players. During childhood and early adolescence, ongoing growth and maturation substantially influence muscular strength, physical performance, and sport-specific skills. As a result, increasing attention has been directed toward identifying sport-specific physical determinants of performance during childhood and early adolescence to support targeted training and long-term athletic development [14]. Previous research in youth racket-sport athletes has shown that age- and sex-related differences in upper-extremity strength become increasingly apparent during development and may contribute to differences in stroke performance [15,16]. In addition to biological maturation, anthropometric characteristics may also influence stroke velocity. Studies in elite junior tennis players have demonstrated that body height, body mass, arm span, and arm length are significantly associated with serve velocity, likely due to the mechanical advantages afforded by larger body dimensions during overhead stroke production [17,18,19]. Consequently, associations observed between strength measures and shuttlecock velocity may be partially explained by developmental and anthropometric factors rather than reflecting independent strength-related contributions. Determining whether specific upper-extremity strength characteristics remain associated with shuttlecock velocity after accounting for such variables may therefore provide more meaningful information for training prescription and talent development.

Despite the recognized importance of shuttlecock velocity, relatively few studies have examined its physical determinants in badminton players. Previous investigations have reported associations between shuttlecock velocity and upper-extremity strength characteristics, including handgrip force, arm muscle strength, and selected shoulder strength measures [3,5,20]. Other studies have primarily focused on biomechanical variables, such as joint moments, segmental positioning, and movement coordination during smash execution [21,22]. Although these studies have improved our understanding of badminton performance, the independent contributions of segmental upper-extremity strength characteristics to shuttlecock velocity remain unclear. In particular, little is known regarding the relative importance of individual upper-extremity muscle groups after accounting for potentially influential factors such as sex and upper-limb length. Addressing this gap may help identify the strength characteristics most closely associated with shuttlecock velocity and provide more targeted information for athlete development and performance enhancement.

Therefore, the aim of this study was to investigate the associations between segmental upper-extremity isometric strength and shuttlecock velocity in young badminton players. In addition, the study examined whether these associations remained significant after controlling for sex and upper-limb length. It was hypothesized that greater upper-extremity strength would be associated with higher shuttlecock velocity and that shoulder-specific strength measures would demonstrate the strongest independent associations with shuttlecock velocity.

## 2. Materials and Methods

### 2.1. Study Design

This cross-sectional study investigated the association between segmental upper-extremity isometric strength and shuttlecock velocity in young badminton players. The study was approved by the Institutional Ethics Committee of Turgut Ozal University (Approval No: 2025/153) and was conducted in accordance with the principles of the Declaration of Helsinki. Written informed consent was obtained from the parents or legal guardians of all participants. Verbal assent was also obtained from all child participants prior to participation.

### 2.2. Participants

An a priori sample size analysis was performed using G*Power software (version 3.1.9.3; Heinrich Heine University Düsseldorf, Germany). Based on a two-tailed correlation analysis and a previously reported effect size of r = 0.67 from a study examining the association between shoulder strength and serve velocity in competitive tennis players [10], with an alpha level of 0.05 and a statistical power of 0.95, the minimum required sample size was estimated to be 22 participants. Accordingly, the final sample of 24 participants exceeded the minimum required sample size for the primary correlation analysis.

Because the subsequent adjusted analyses involved multiple linear regression models with three predictors, an additional sensitivity analysis was performed using G*Power. With a total sample size of 24, three predictors, and α = 0.05, the analysis indicated that the regression models could detect an overall effect size of f^2^ ≥ 0.55 with 80% power and f^2^ ≥ 0.72 with 90% power. Thus, the multivariable analyses were primarily sensitive to large effects.

Twenty-four competitive badminton players (12 males and 12 females) competing in the Under-11 (U11) age category participated in the study. All participants were 10 years of age and had a minimum of two years of badminton experience. All participants were actively engaged in regular badminton training and competition, with at least two years of experience. Participants were recruited from local badminton clubs and regularly competed in age-group tournaments. Inclusion criteria were: (1) competing in the Under-11 (U11) badminton age category, (2) having at least two years of badminton training experience, and (3) regular participation in organized badminton training and competition. Participants were excluded if they had any musculoskeletal injury affecting the upper extremity within the previous six months, any neurological disorder, or any medical condition that could influence strength or performance testing.

**Figure 1:**
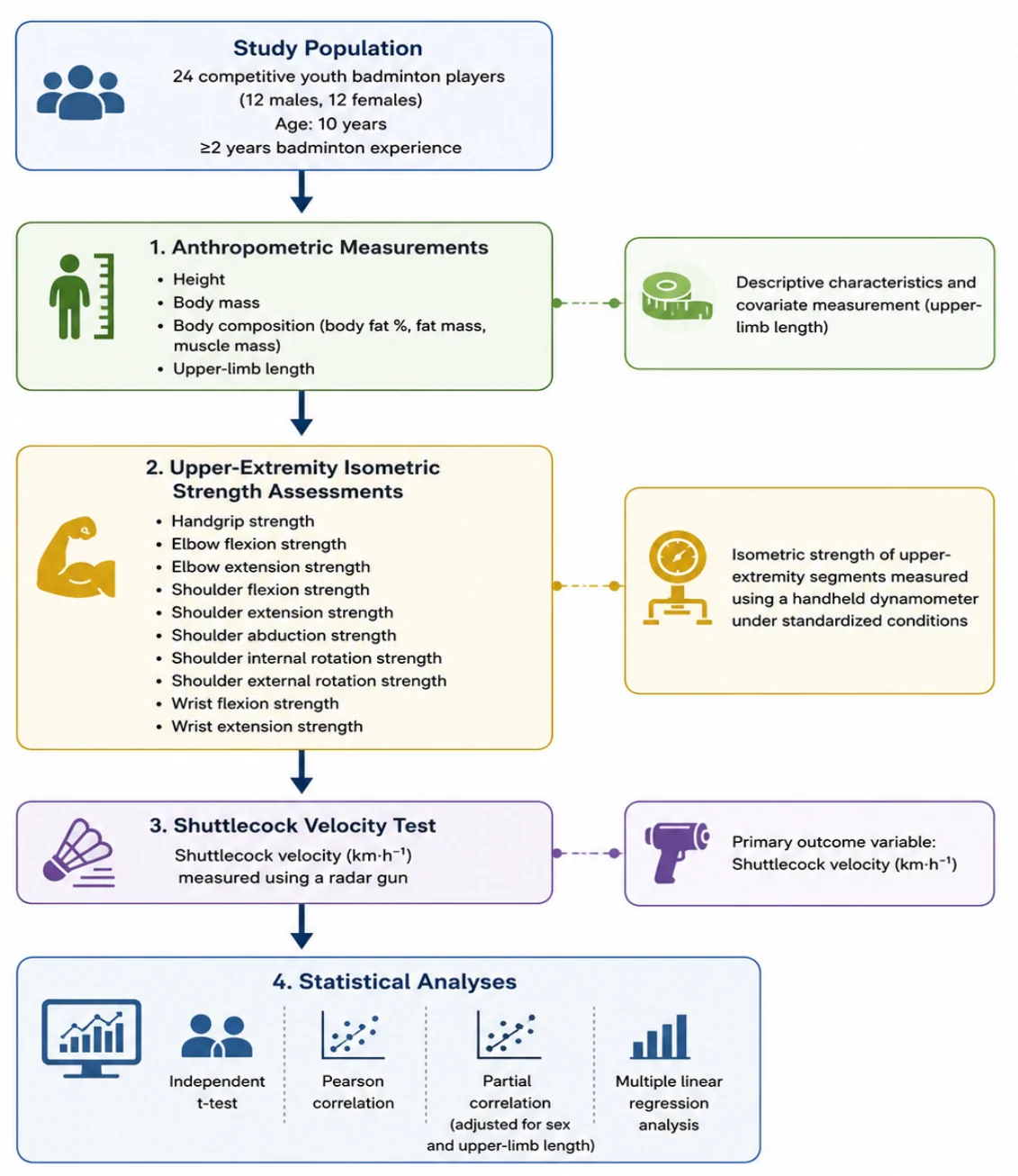
Summary of the study design, assessment procedures, and statistical analyses.

Anthropometric measurements, upper-limb length assessments, upper-extremity isometric strength testing, and shuttlecock velocity assessments were subsequently performed under standardized conditions, as illustrated in Figure 1.

### 2.3. Body Composition and Anthropometric Measurements

Body composition and anthropometric assessments were performed prior to strength and shuttlecock velocity testing. Body height was measured using a wall-mounted stadiometer (seca 213, seca GmbH & Co. KG, Hamburg, Germany) to the nearest 0.1 cm, and body mass was measured to the nearest 0.1 kg with participants wearing light clothing and no shoes.

Body composition variables, including body fat percentage, fat mass, and muscle mass, were assessed using a bioelectrical impedance analyzer (Tanita SC-330S, Tokyo, Japan) according to the manufacturer’s guidelines. Body mass index (BMI) was subsequently calculated as body mass (kg) divided by height squared (m^2^).

Upper-limb length was measured with a standard measuring tape while participants stood in an anatomical position. The distance between the acromion process and the tip of the middle finger of the dominant upper extremity was recorded to the nearest 0.1 cm. All anthropometric measurements were performed by the same examiner under standardized conditions.

### 2.4. Upper-Extremity Isometric Strength Assessment

Upper-extremity strength assessments were performed using standardized dynamometry procedures. Handgrip strength was measured using a Jamar Hand Dynamometer (Patterson Medical, Warrenville, IL, USA), whereas segmental upper-extremity isometric strength, including elbow flexion and extension, shoulder flexion, extension, abduction, internal rotation and external rotation, and wrist flexion and extension strength, was assessed using a Lafayette Manual Muscle Tester (Model 01165, Lafayette Instrument Company, Lafayette, IN, USA). Testing positions and dynamometer placements were adapted from previously published handheld dynamometry protocols for upper-extremity strength assessment [23,24,25,26,27]. Handgrip strength was assessed according to the recommendations of the American Society of Hand Therapists (ASHT) [28] (Table 1).

Prior to testing, participants completed a standardized warm-up consisting of light dynamic upper-extremity movements and submaximal practice contractions. Subsequently, three maximal voluntary isometric contractions were performed for each strength assessment, with a 30 s rest interval between trials to minimize fatigue. Standardized verbal encouragement was provided throughout testing, and the mean value of the three trials was used for subsequent analyses. The intra-rater reliability of the strength measurements was evaluated using intraclass correlation coefficients (ICC; two-way mixed-effects model, absolute agreement, average measures). ICC values exceeded 0.90 for all strength assessments, indicating excellent reliability.

### 2.5. Shuttlecock Velocity Assessment

Shuttlecock velocity was assessed during a badminton smash using a calibrated radar gun (Bushnell Speedster Radar Gun, Model 101911, Bushnell Corporation, Overland Park, KS, USA). Prior to testing, participants completed a standardized badminton-specific warm-up consisting of light rallying, dynamic upper-extremity movements, and progressive practice smashes.

Participants were instructed to perform maximal-effort forehand smashes directed toward the opponent’s court under standardized testing conditions. Three valid trials were completed, with sufficient recovery provided between attempts to minimize fatigue. Shuttlecock velocity was recorded in kilometers per hour (km·h^−1^), and the mean value of the three trials was used for subsequent analyses. To ensure measurement consistency, all assessments were conducted by the same examiner using identical testing procedures and equipment throughout the study.

### 2.6. Statistical Analysis

Statistical analyses were performed using IBM SPSS Statistics software (Version 24; IBM Corporation, Armonk, NY, USA). Data were screened for completeness, outliers, and distributional assumptions prior to analysis. The normality of continuous variables was evaluated using the Shapiro–Wilk test together with visual inspection of histograms and Q–Q plots. Descriptive statistics are presented as mean ± standard deviation (SD). Sex-based differences in anthropometric characteristics, upper-extremity strength variables, and shuttlecock velocity were examined using independent-samples *t*-tests. Effect sizes were calculated using Cohen’s d and interpreted as trivial (<0.20), small (0.20–0.49), moderate (0.50–0.79), and large (≥0.80). Pearson product–moment correlation coefficients were calculated to examine the associations between shuttlecock velocity and upper-extremity strength variables. Given the exploratory nature of the study and the limited prior evidence regarding segment-specific strength–velocity associations in young badminton players, the correlation and subsequent regression analyses were considered exploratory and hypothesis-generating. Therefore, no formal adjustment for multiple comparisons was applied, and the reported *p*-values should be interpreted within this exploratory framework. Because all participants were 10 years of age, chronological age showed no between-participant variability and was therefore not included as a covariate. Other measured anthropometric variables were examined; however, to maintain a parsimonious model and limit overfitting given the modest sample size, sex and upper-limb length were retained as the principal covariates. To account for the potential influence of sex and upper-limb length, partial correlation analyses were subsequently performed while controlling for these variables. Strength variables that remained significantly associated with shuttlecock velocity in the partial correlation analyses were entered into separate multiple linear regression models. Shuttlecock velocity was specified as the dependent variable, whereas sex, upper-limb length, and the respective strength variable were included as independent variables. Standardized regression coefficients (β), 95% confidence intervals (CIs), and coefficients of determination (R^2^) were reported. Multicollinearity was assessed using the variance inflation factor (VIF) values and tolerance statistics. The reliability of strength measurements was evaluated using intraclass correlation coefficients (ICC; two-way mixed-effects model, absolute agreement, average measures). All reported *p*-values were two-sided, with an unadjusted significance level of *p* < 0.05.

## 3. Results

Participant characteristics according to sex are presented in Table 2. No significant differences were observed between male and female players for height, body mass, body mass index, body fat percentage, fat mass, muscle mass, or upper-limb length (all *p* > 0.05). Effect size analyses indicated trivial-to-small differences between the sexes across all anthropometric variables (Cohen’s d = 0.04–0.35), suggesting that the two groups were comparable in terms of anthropometry.

Sex-based comparisons of upper-extremity strength variables and shuttlecock velocity are presented in Table 3. Male players demonstrated significantly greater elbow flexion strength (*p* = 0.004, d = 1.30), shoulder abduction strength (*p* = 0.017, d = 1.05), and shoulder internal rotation strength (*p* = 0.017, d = 1.07) than female players. In addition, male players achieved significantly higher shuttlecock velocities than female players (*p* < 0.001, d = 1.76). The observed effect sizes for these variables were large to very large.

No significant sex differences were observed for handgrip strength, elbow extension strength, shoulder extension strength, shoulder flexion strength, shoulder external rotation strength, wrist extension strength, or wrist flexion strength (all *p* > 0.05). Nevertheless, shoulder external rotation strength demonstrated a large effect size (d = 0.85) despite not reaching statistical significance.

Pearson correlation analyses demonstrated significant positive associations between shuttlecock velocity and all measured upper-extremity strength variables (*r* = 0.408–0.704, all *p* < 0.05). However, after controlling for sex and upper-limb length, only shoulder extension strength (*r* = 0.529, *p* = 0.011), shoulder flexion strength (*r* = 0.557, *p* = 0.007), shoulder external rotation strength (*r* = 0.430, *p* = 0.046), and shoulder internal rotation strength (*r* = 0.495, *p* = 0.019) remained significantly associated with shuttlecock velocity. In contrast, the associations observed for handgrip, elbow, shoulder abduction, and wrist strength measures were no longer statistically significant following adjustment (Table 4).

**Figure 2:**
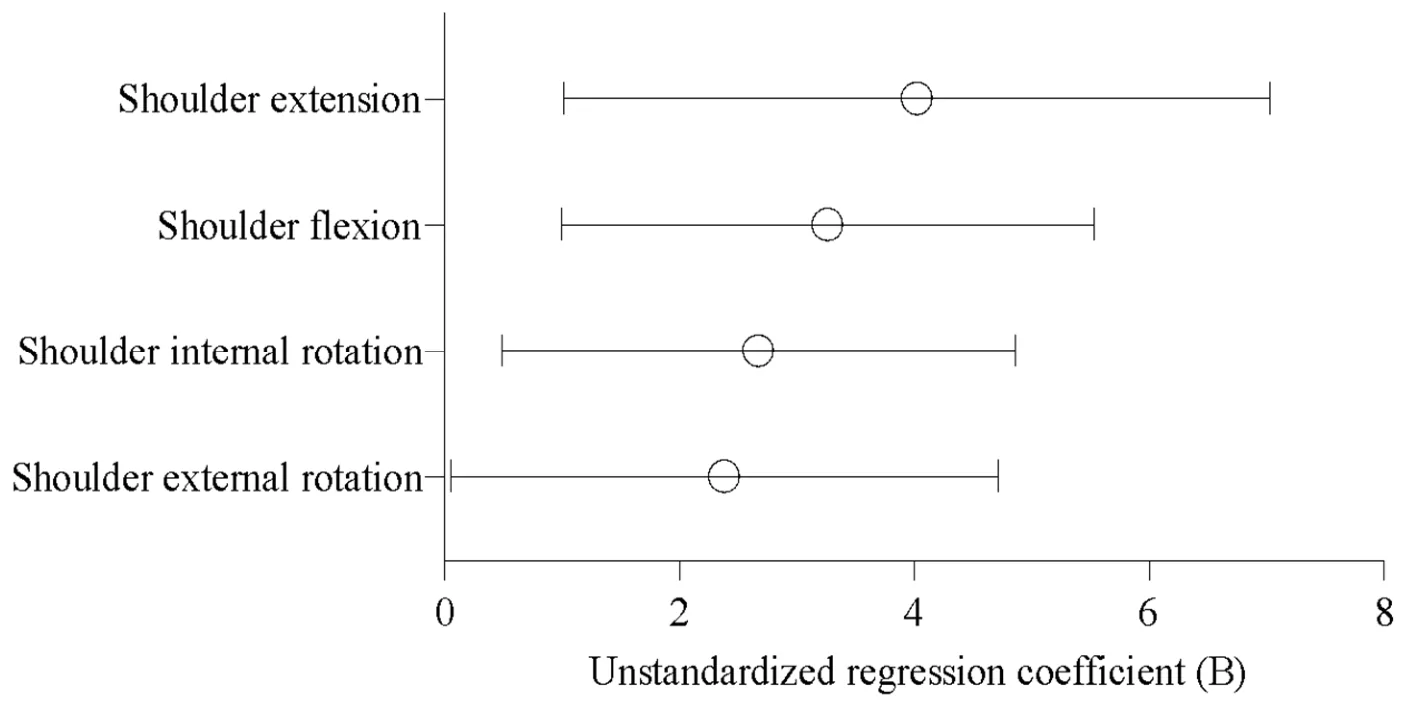
Unstandardized regression coefficients (B) and 95% confidence intervals for shoulder-specific strength variables independently associated with shuttlecock velocity after adjustment for sex and upper-limb length.

Multiple linear regression analyses were conducted to further examine the associations between shoulder-specific strength variables and shuttlecock velocity, while controlling for sex and upper-limb length (Table 5). Shoulder flexion strength (β = 0.463, *p* = 0.007), shoulder extension strength (β = 0.468, *p* = 0.011), shoulder external rotation strength (β = 0.343, *p* = 0.046), and shoulder internal rotation strength (β = 0.424, *p* = 0.019) were independently associated with shuttlecock velocity. Consistent with the partial correlation analyses, shoulder-specific strength measures remained significant after adjustment for potential confounding variables. The regression models demonstrated substantial explanatory power, accounting for 62.8–68.5% of the variance in shuttlecock velocity (R^2^ = 0.628–0.685). Corresponding adjusted R^2^ values ranged from 0.572 to 0.638. Across all models, upper-limb length was not a significant predictor of shuttlecock velocity, whereas sex remained a significant predictor. Figure 2 visually summarizes the magnitude and precision of the regression coefficients for the shoulder-specific strength variables.

## 4. Discussion

The present study investigated the associations between segmental upper-extremity isometric strength and shuttlecock velocity in young badminton players. The principal finding was that although shuttlecock velocity was positively associated with all measured upper-extremity strength variables in the initial correlation analyses, only shoulder flexion, shoulder extension, shoulder internal rotation, and shoulder external rotation strength remained significantly associated with shuttlecock velocity after controlling for sex and upper-limb length. Furthermore, these shoulder-specific strength variables independently predicted shuttlecock velocity in the regression analyses, whereas upper-limb length was not a significant predictor in any model. Collectively, these findings suggest that shoulder strength may play a particularly important role in shuttlecock velocity generation in young badminton players beyond the influence of general upper-extremity strength and anthropometric characteristics.

The substantial attenuation of the strength–velocity relationships following statistical adjustment is consistent with evidence indicating that growth, maturation, and anthropometric characteristics substantially influence physical performance in youth athletes. Advanced biological maturity has been associated with greater upper-extremity strength and sport-specific performance in youth racket-sport athletes [16], while age- and sex-related differences in physical capacities have also been reported in junior badminton players [29]. Moreover, previous studies have shown that initially significant associations between physical characteristics and performance outcomes may diminish after controlling for maturation- and anthropometry-related factors [30,31]. Consistent with these observations, significant correlations involving handgrip, elbow, and wrist strength were no longer evident after adjustment for sex and upper-limb length in the present study. In contrast, shoulder-specific strength variables remained significant, suggesting a more robust relationship with shuttlecock velocity that is less dependent on developmental and anthropometric influences.

The persistence of shoulder flexion, extension, internal rotation, and external rotation strength after adjustment is biomechanically plausible given the central role of the shoulder complex during badminton stroke production. Consistent with the present findings, previous studies have reported significant associations between shoulder strength and performance in overhead sports. Awatani et al. [32] observed a significant relationship between shoulder internal rotation strength and racket velocity during the badminton forehand smash (*r* = 0.652), while Baiget et al. [10] reported that shoulder internal rotation and flexion strength together explained 55% of the variance in tennis serve velocity. Similarly, force-producing characteristics of shoulder internal rotation and flexion have been identified as important determinants of serve performance in high-level tennis players [11]. From a biomechanical perspective, shoulder internal rotation is considered one of the primary contributors to racket-head acceleration during overhead striking actions, accounting for approximately 66% of racket-head speed generation in some models [33]. Furthermore, kinematic analyses of the badminton smash have consistently highlighted the importance of shoulder internal and external rotation velocities during the acceleration and maximal external rotation phases of the stroke [8]. Together, these findings support the notion that shoulder-specific strength may play a fundamental role in the efficient transfer of mechanical energy through the proximal-to-distal kinetic chain, thereby contributing to greater shuttlecock velocity.

Although shoulder internal rotation demonstrated the strongest unadjusted association with shuttlecock velocity, shoulder flexion, extension, internal rotation, and external rotation strength all remained significant following adjustment. This pattern suggests that shuttlecock velocity is unlikely to depend on a single shoulder action. Rather, performance appears to be influenced by the coordinated contribution of multiple shoulder movement patterns operating across different phases of the smash. This interpretation is supported by recent electromyographic and muscle-synergy investigations demonstrating that badminton smash performance relies on the integrated activation of several shoulder-related muscle groups rather than isolated muscle actions. Tajik et al. [34] reported that a muscle synergy dominated by the pectoralis complex and anterior deltoid contributes primarily to shoulder flexion and internal rotation during the acceleration and impact phases of the smash, whereas a separate synergy involving the middle deltoid and posterior musculature supports shoulder extension and external rotation. Similarly, Zhang [35] identified the pectoralis major and anterior and posterior deltoid muscles as the most highly activated muscle groups during the badminton forehand smash, while Fong et al. [36] reported particularly high activation of the pectoralis major, anterior deltoid, infraspinatus, and latissimus dorsi during smash performance. Collectively, these findings indicate that successful shuttlecock velocity generation depends on the coordinated interaction of multiple shoulder muscle groups across the preparation, acceleration, and follow-through phases of the smash, which may explain why several shoulder-specific strength variables independently contributed to performance in the present study.

The regression models’ relatively high explanatory power further supports the practical relevance of shoulder-specific strength in youth badminton players. In the present study, shoulder strength variables explained approximately 63–69% of the variance in shuttlecock velocity, even after accounting for sex and upper-limb length. Comparable findings have been reported in racket sports, where shoulder-related strength characteristics have consistently emerged as important predictors of stroke performance. Baiget et al. [10] reported that shoulder flexion and internal rotation strength explained 55% of the variance in tennis serve velocity, whereas Fernández-Fernández et al. [15] observed that models incorporating shoulder strength measures explained up to 71.3% of serve-speed variance in young tennis players. Similarly, physical performance models including upper-extremity strength variables explained between 41% and 66% of serve velocity in elite junior tennis players [17], while Bilić et al. [37] and Stubbe et al. [38] reported explanatory values ranging from approximately 59% to 75%. Taken together, these findings suggest that upper-extremity strength characteristics, particularly those involving the shoulder complex, account for a substantial proportion of stroke-velocity performance across racket sports. From a practical perspective, shoulder-strength assessments may provide potentially useful information for athlete monitoring; however, the present findings alone do not support specific training prescriptions. Furthermore, shoulder-specific isometric strength training has been shown to improve serve velocity in young tennis players [11], suggesting that the shoulder strength characteristics identified in the present study may warrant further investigation as potentially modifiable performance-related qualities.

Several limitations should be considered when interpreting the present findings. First, the cross-sectional design precludes causal inference. Although shoulder-specific strength was associated with shuttlecock velocity, it cannot be concluded that increases in shoulder strength will necessarily result in improvements in shuttlecock velocity. Second, the sample consisted exclusively of 10-year-old badminton players, limiting the generalizability of the findings to athletes of different ages, competitive levels, and stages of biological maturation. Moreover, although chronological age was homogeneous across the sample, biological maturation was not directly assessed; therefore, potential inter-individual differences in maturational status and their influence on strength and performance could not be accounted for. Third, although sex and upper-limb length were controlled statistically, other potentially relevant factors, including technical proficiency, lower-limb strength, trunk strength, and training volume, were not evaluated. In addition, the modest sample size warrants caution when interpreting the multivariable findings. The effect size used for the a priori sample size calculation was derived from adult competitive tennis players and may not accurately represent the strength–velocity relationship in 10-year-old badminton players. Sensitivity analysis indicated that the regression models were primarily sensitive to large effects. With 24 observations and three predictors per model (8 observations per predictor; 6 observations per estimated regression coefficient when the intercept was included), the modest observation-to-parameter ratio, together with the relatively wide confidence intervals for some coefficients, may increase the risk of coefficient instability and overfitting. Furthermore, multiple strength variables were examined without formal adjustment for multiple comparisons. Accordingly, the correlation and regression analyses should be regarded as exploratory and hypothesis-generating, and an increased risk of Type I error and false-positive associations cannot be excluded. These findings therefore require cautious interpretation and confirmation in larger, adequately powered samples. Finally, only isometric strength measures were examined. Given the dynamic and explosive nature of badminton stroke production, future studies incorporating longitudinal designs together with biomechanical, neuromuscular, and power-related assessments may provide a more comprehensive understanding of the determinants of shuttlecock velocity.

Nevertheless, the present study provides novel insights into the physical correlates of shuttlecock velocity in young badminton players. To our knowledge, this is one of the first studies to examine segmental upper-extremity strength across multiple joint actions while simultaneously accounting for important confounding factors such as sex and upper-limb length. The findings demonstrate that shoulder-specific strength characteristics remain associated with shuttlecock velocity even after adjustment for these variables, highlighting the potential importance of the shoulder complex in youth badminton performance. These observations may contribute to a more targeted approach to athlete assessment and conditioning within long-term player development programs.

## 5. Conclusions

The present exploratory study found that although shuttlecock velocity was initially associated with a broad range of upper-extremity strength measures, shoulder flexion, extension, internal rotation, and external rotation strength remained associated with performance after accounting for sex and upper-limb length. These findings suggest that shoulder-specific strength may represent a relevant physical correlate of badminton smash performance in young athletes. However, given the small, single-site sample and the exploratory nature of the analyses, these associations should be interpreted cautiously and should not be considered evidence that targeted shoulder-strength training will improve shuttlecock velocity. Larger, adequately powered prospective studies are needed to confirm these findings and determine whether improvements in shoulder strength translate into meaningful enhancements in shuttlecock velocity and on-court performance.

## Figures and Tables

**Table 1:** Standardized testing positions and dynamometer placement for upper-extremity isometric strength assessments.

Measurement	Participant Position	Dynamometer Placement
Handgrip strength	Seated, shoulder adducted and neutral, elbow flexed to 90°, forearm neutral, wrist 0–30° extension	Hand dynamometer held in the dominant hand
Elbow flexion strength	Seated, shoulder neutral, elbow flexed to 90°, forearm supinated	Volar distal forearm, proximal to the wrist
Elbow extension strength	Seated, shoulder neutral, elbow flexed to 90°, forearm supinated	Dorsal distal forearm, proximal to the wrist
Shoulder flexion strength	Seated, shoulder in neutral position, elbow extended	Distal posterior humerus, proximal to the elbow
Shoulder extension strength	Seated, shoulder in neutral position, elbow extended	Distal lateral humerus, proximal to the elbow
Shoulder abduction strength	Seated, shoulder abducted to 90°, elbow extended	Distal humerus
Shoulder internal rotation strength	Seated, shoulder neutral, elbow flexed to 90°, forearm neutral	Volar distal forearm
Shoulder external rotation strength	Seated, shoulder neutral, elbow flexed to 90°, forearm neutral	Dorsal distal forearm
Wrist flexion strength	Seated, forearm supinated and supported, wrist neutral	Palmar aspect of the hand, over the proximal metacarpals
Wrist extension strength	Seated, forearm pronated and supported, wrist neutral	Dorsal aspect of the hand, over the proximal metacarpals

**Table 2:** Participant Characteristics According to Sex.

Variable	Male	Female	Mean Difference	t	*p*	ES
Height (cm)	135.25 ± 8.28	138.33 ± 9.46	−3.08	−0.850	0.405	0.35
Body mass (kg)	31.82 ± 9.83	32.21 ± 12.351	−0.39	−0.086	0.932	0.04
Body mass index (kg/m^2^)	17.16 ± 4.00	16.51 ± 4.94	0.64	0.353	0.727	0.14
Body fat (%)	16.88 ± 8.38	19.37 ±9.52	−2.49	−0.680	0.503	0.28
Fat mass (kg)	6.06 ± 5.28	7.17 ± 5.97	−1.10	−0.481	0.635	0.20
Muscle mass (kg)	24.33 ± 4.75	23.72 ± 6.71	0.60	0.256	0.800	0.10
Upper limb length (cm)	60.63 ± 6.84	61.41 ± 5.22	−0.78	−0.315	0.756	0.13

Values are presented as mean ± standard deviation (SD). Sex-based comparisons were performed using independent-samples *t*-tests. ES = effect size (Cohen’s d). *p* < 0.05.

**Table 3:** Sex-Based Comparisons of Upper-Extremity Strength and Shuttlecock Velocity.

Variable	Male	Female	Mean Difference	t	*p*	ES
Handgrip strength (kg)	14.16 ± 3.12	12.61 ± 4.02	1.55	1.053	0.304	0.43
Elbow extension strength (kg)	9.76 ± 2.69	8.22 ± 1.47	1.54	1.736	0.090	0.71
Elbow flexion strength (kg)	12.40 ± 2.61	9.56 ± 1.61	2.83	3.193	0.004	1.30
Shoulder extension strength (kg)	8.48 ± 1.20	7.65 ± 2.20	0.83	1.151	0.262	0.47
Shoulder flexion strength (kg)	9.85 ± 1.23	9.01 ± 2.82	0.84	0.935	0.360	0.38
Shoulder abduction strength (kg)	7.50 ± 0.77	6.53 ± 1.05	0.97	2.572	0.017	1.05
Shoulder external rotation strength (kg)	9.42 ± 2.65	7.66 ± 1.22	1.75	2.084	0.054	0.85
Shoulder internal rotation strength (kg)	10.96 ± 2.66	8.63 ± 1.52	2.33	2.627	0.017	1.07
Wrist extension strength (kg)	9.03 ± 2.51	7.74 ± 1.93	1.29	1.412	0.172	0.58
Wrist flexion strength (kg)	10.96 ± 2.78	9.48 ± 1.87	1.48	1.531	0.140	0.62
Shuttlecock velocity (km·h^−1^)	82.91 ± 7.63	62.58 ± 14.45	20.33	4.309	<0.001	1.76

Values are presented as mean ± standard deviation (SD). Sex-based comparisons were performed using independent-samples *t*-tests. ES, effect size (Cohen’s d). *p* < 0.05.

**Table 4:** Pearson and Partial Correlations Between Upper-Extremity Strength Variables and Shuttlecock Velocity.

Variable	Pearson *r*	*p*	Partial r ^†^	*p*
Handgrip strength (kg)	0.440	0.031	0.205	0.360
Elbow extension strength (kg)	0.595	0.002	0.391	0.072
Elbow flexion strength (kg)	0.684	<0.001	0.395	0.069
Shoulder extension strength (kg)	0.610	0.002	0.529	0.011
Shoulder flexion strength (kg)	0.599	0.002	0.557	0.007
Shoulder abduction strength (kg)	0.451	0.027	0.074	0.742
Shoulder external rotation strength (kg)	0.621	0.001	0.430	0.046
Shoulder internal rotation strength (kg)	0.704	<0.001	0.495	0.019
Wrist extension strength (kg)	0.412	0.045	0.250	0.262
Wrist flexion strength (kg)	0.408	0.048	0.215	0.337

^†^ = Partial correlations adjusted for sex and upper-limb length. Pearson *r* = Pearson correlation coefficient; Partial *r* = partial correlation coefficient.

**Table 5:** Multiple Linear Regression Models Examining the Associations Between Shoulder-Specific Strength and Shuttlecock Velocity.

Variable	B	95% CI	β	*p*	R^2^	Adj R^2^
Sex	−17.007	−25.585, −8.429	−0.566	0.001	0.671	0.621
Upper-limb length (cm)	0.031	−0.846, 0.907	0.012	0.943		
Shoulder extension strength (kg)	4.021	1.011, 7.030	0.468	0.011		
Sex	−17.693	−25.895, −9.492	−0.589	<0.001	0.685	0.638
Upper-limb length (cm)	0.100	−0.714, 0.914	0.039	0.800		
Shoulder flexion strength (kg)	3.262	0.994, 5.530	0.463	0.007		
Sex	−16.498	−26.103, −6.894	−0.549	0.002	0.628	0.572
Upper-limb length (cm)	0.451	−0.340, 1.242	0.175	0.248		
Shoulder external rotation strength (kg)	2.382	0.052, 4.712	0.343	0.046		
Sex	−14.373	−24.209, −4.536	−0.478	0.006	0.655	0.604
Upper-limb length (cm)	0.347	−0.434, 1.127	0.135	0.365		
Shoulder internal rotation strength (kg)	2.671	0.487, 4.855	0.424	0.019		

B = unstandardized regression coefficient; β = standardized regression coefficient; CI = confidence interval; R^2^ = coefficient of determination; Adj. R^2^ = adjusted coefficient of determination. Sex and upper-limb length were included as covariates in all models.

## Data Availability

The data that support the findings of this study are available from the corresponding author upon reasonable request.

## Abbreviations

The following abbreviations are used in this manuscript:

ASHT	American Society of Hand Therapists
U11	Under-11

## References

[1] Phomsoupha M., Laffaye G. (2014). Shuttlecock velocity during a smash stroke in badminton evolves linearly with skill level. Comput. Methods Biomech. Biomed. Eng..

[2] Phomsoupha M., Laffaye G. (2015). The science of badminton: Game characteristics, anthropometry, physiology, visual fitness and biomechanics. Sports Med..

[3] Ferreira A., Górski M., Gajewski J. (2020). Gender differences and relationships between upper extremity muscle strength, lower limb power and shuttle velocity in forehand smash and jump smash in badminton. Acta Bioeng. Biomech..

[4] Le Mansec Y., Perez J., Rouault Q., Doron J., Jubeau M. (2020). Impaired performance of the smash stroke in badminton induced by muscle fatigue. Int. J. Sports Physiol. Perform..

[5] Phomsoupha M., Laffaye G. (2020). Multiple repeated-sprint ability test with four changes of direction for badminton players (Part 2): Predicting skill level with anthropometry, strength, shuttlecock, and displacement velocity. J. Strength Cond. Res..

[6] Zhang Z., Li S., Wan B., Visentin P., Jiang Q., Dyck M., Shan G. (2016). The influence of X-factor (trunk rotation) and experience on the quality of the badminton forehand smash. J. Hum. Kinet..

[7] Li F., Li S., Zhang X., Shan G. (2023). Biomechanical insights for developing evidence-based training programs: Unveiling the kinematic secrets of the overhead forehand smash in badminton through novice-skilled player comparison. Appl. Sci..

[8] Rusdiana A., Abdullah M.R.B., Syahid A.M., Haryono T., Kurniawan T. (2021). Badminton overhead backhand and forehand smashes: A biomechanical analysis approach. J. Phys. Educ. Sport.

[9] King M., Towler H., Dillon R., McErlain-Naylor S. (2020). A correlational analysis of shuttlecock speed kinematic determinants in the badminton jump smash. Appl. Sci..

[10] Baiget E., Corbi F., Fuentes J.P., Fernández-Fernández J. (2016). The relationship between maximum isometric strength and ball velocity in the tennis serve. J. Hum. Kinet..

[11] Baiget E., Colomar J., Corbi F. (2021). Upper-limb force–time characteristics determine serve velocity in competition tennis players. Int. J. Sports Physiol. Perform..

[12] Ölmez C., Hammami N., Apaydın N., Hattabi S., Şar H., Khezami M.A., İnce A. (2025). Is isokinetic shoulder strength a determinant of serve ball velocity in tennis?. Sports Biomech..

[13] Parpa K., Michaelides M., Petrov D., Kyrillou C., Paludo A.C. (2022). Relationship between physical performance, anthropometric measurements and stroke velocity in youth tennis players. Sports.

[14] Aydin Y., Tokgoz G., Yilmaz N., Coskun I.A., Beykumul A., Colak E., Aygoren C., Koc S., Cinarli F.S. (2025). Assessing motor performance and ankle mobility in pre-adolescent male fencers. Life.

[15] Fernandez-Fernandez J., Nakamura F.Y., Moreno-Perez V., Lopez-Valenciano A., Del Coso J., Gallo-Salazar C., Sanz-Rivas D. (2019). Age- and sex-related upper body performance differences in competitive young tennis players. PLoS ONE.

[16] Myburgh G.K., Cumming S.P., Silva M.C.E., Cooke K., Malina R.M. (2016). Maturity-associated variation in functional characteristics of elite youth tennis players. Pediatr. Exerc. Sci..

[17] Fett J., Ulbricht A., Ferrauti A. (2020). Impact of physical performance and anthropometric characteristics on serve velocity in elite junior tennis players. J. Strength Cond. Res..

[18] Ma X., Yaacob A., Kamalden T.F.T., Ng Y.G. (2024). To assess the impact of physical factors on velocity, speed, and accuracy of tennis serve. J. Nat. Sci. Biol. Med..

[19] Baiget E., Corbi F., López J. (2023). Influence of anthropometric, ball impact and landing location parameters on serve velocity in elite tennis competition. Biol. Sport.

[20] Purnama A.R., Kusdinar Y., Kardjono K. (2024). The relationship between wrist flexibility and arm muscle strength with the accuracy and speed of smash for badminton athletes Universitas Pendidikan Indonesia (UPI). J. Phys. Educ. Health Sport.

[21] Ramasamy Y., Sundar V., Usman J., Razman R., Towler H., King M.A. (2022). Relationships between racket arm joint moments and racket head speed during the badminton jump smash performed by elite male Malaysian players. Appl. Sci..

[22] Ramasamy Y., Usman J., Sundar V., Towler H., King M. (2024). Kinetic and kinematic determinants of shuttlecock speed in the forehand jump smash performed by elite male Malaysian badminton players. Sports Biomech..

[23] Kilmer D.D., McCrory M.A., Wright N.C., Rosko R.A., Kim H.R., Aitkens S.G. (1997). Hand-held dynamometry reliability in persons with neuropathic weakness. Arch. Phys. Med. Rehabil..

[24] Daloia L.M.T., Leonardi-Figueiredo M.M., Martinez E.Z., Mattiello-Sverzut A.C., Carvalho M.D.M. (2018). Isometric muscle strength in children and adolescents using handheld dynamometry: Reliability and normative data for the Brazilian population. Braz. J. Phys. Ther..

[25] Westrick R.B., Duffey M.L., Cameron K.L., Gerhardt M.B., Owens B.D., Taylor D.C. (2013). Isometric shoulder strength reference values for physically active collegiate males and females. Sports Health.

[26] Karagiannopoulos C., Griech S., McFarland C., Korakakis V. (2022). Reliability and validity of the ActivForce digital dynamometer in assessing shoulder muscle force across different user experience levels. Int. J. Sports Phys. Ther..

[27] Bradley H., Pierpoint L. (2023). Normative values of isometric shoulder strength among healthy adults. Int. J. Sports Phys. Ther..

[28] Sousa-Santos A.R., Amaral T.F. (2017). Differences in handgrip strength protocols to identify sarcopenia and frailty—A systematic review. BMC Geriatr..

[29] Winata B., Brochhagen J., Apriantono T., Hoppe M.W. (2025). Do anthropometric characteristics and physical capacities of highly trained junior badminton players differ according to age and sex?. Front. Sports Act. Living.

[30] Abbott S., Leong W.E., Gwinn T., Postiglione G.L., Salter J., Cobley S. (2022). The longitudinal mediating influence of maturation on the relationship between strength and performance in male youth swimmers. Int. J. Sports Physiol. Perform..

[31] González-González I., Rodríguez-Rosell D., Clavero-Martín D., Mora-Custodio R., Pareja-Blanco F., García J.M.Y., González-Badillo J.J. (2018). Reliability and accuracy of ball speed during different strokes in young tennis players. Sports Med. Int. Open.

[32] Awatani T., Morikita I., Urata T., Shinohara J., Tatsumi Y. (2018). Correlation between isometric shoulder strength and racket velocity during badminton forehand smash movements: Study of valid clinical assessment methods. J. Phys. Ther. Sci..

[33] Ramasamy Y., Usman J., Razman R., Wei Y.M., Towler H., King M. (2023). A systematic review of the biomechanical studies on shoulder kinematics in overhead sporting motions: Types of analysis and approaches. Appl. Sci..

[34] Tajik R., Dhahbi W., Fadaei H., Mimar R. (2025). Muscle synergy analysis during badminton forehand overhead smash: Integrating electromyography and musculoskeletal modeling. Front. Sports Act. Living.

[35] Zhang C. (2021). Characteristics of surface electromyography of forehand smash of badminton players. Mol. Cell. Biomech..

[36] Fong S.M., Ng L.K., Ma W.W., Wang H.K., Bae Y.H., Yam T.T., Kam W.K., Chung W.Y. (2019). Effects of kinesiology taping on shoulder girdle muscle activity and sports performance during badminton forehand overhead strokes in amateur badminton players with shoulder impingement syndrome. J. Sports Med. Phys. Fit..

[37] Bilić Z., Martić P., Barbaros P., Sinković F., Novak D. (2024). Neuromuscular fitness is associated with serve speed in young female tennis players. Sports.

[38] Stubbe J., Borms D., Vandenbosch D., Cools A.M. (2026). Validation of an upper extremity physical performance test battery in competitive adult tennis players. Sports Health.

